# Ecological interactions driving population dynamics of two tick-borne pathogens, *Borrelia burgdorferi* and *Babesia microti*

**DOI:** 10.1098/rspb.2023.0642

**Published:** 2023-06-28

**Authors:** Danielle M. Tufts, Ben Adams, Maria A. Diuk-Wasser

**Affiliations:** ^1^ Infectious Diseases and Microbiology, University of Pittsburgh, Pittsburgh, PA, USA; ^2^ Department of Veterinary Tropical Diseases, University of Pretoria, Pretoria, South Africa; ^3^ Ecology, Evolution, and Environmental Biology, Columbia University, New York, NY, USA; ^4^ Department of Mathematical Sciences, University of Bath, Bath, UK

**Keywords:** blacklegged tick, white-footed mouse, reservoir host, vertical transmission, eco-epidemiological model, mechanistic model

## Abstract

*Borrelia burgdorferi* (*Bb*) and *Babesia microti* (*Bm*) are vector-borne zoonotic pathogens commonly found co-circulating in *Ixodes scapularis* and *Peromyscus leucopus* populations. The restricted distribution and lower prevalence of *Bm* has been historically attributed to lower host-to-tick transmission efficiency and limited host ranges. We hypothesized that prevalence patterns are driven by coinfection dynamics and vertical transmission. We use a multi-year, multiple location, longitudinal dataset with mathematical modelling to elucidate coinfection dynamics between *Bb* and *Bm* in natural populations of *P. leucopus*, the most competent reservoir host for both pathogens in the eastern USA. Our analyses indicate that, in the absence of vertical transmission, *Bb* is viable at lower tick numbers than *Bm.* However, with vertical transmission, *Bm* is viable at lower tick numbers than *Bb*. Vertical transmission has a particularly strong effect on *Bm* prevalence early in the active season while coinfection has an increasing role during the nymphal peak. Our analyses indicate that coinfection processes, such as facilitation of *Bm* infection by *Bb*, have relatively little influence on the persistence of either parasite. We suggest future work examines the sensitivity of *Bm* vertical transmission and other key processes to local environmental conditions to inform surveillance and control of tick-borne pathogens.

## 1. Introduction

Understanding the ecological factors driving the distribution and abundance of zoonotic pathogens is vital for assessing and predicting the risk of spillover into human populations. The ability for zoonotic pathogens to invade and persist in new populations is determined by their capacity to successfully infect and be transmitted to one or more hosts such that the basic reproductive number *R*_0_ > 1 [1,2]. For vector-borne diseases, most studies investigate the vector or host and environmental drivers of individual pathogens. However, coinfections tend to be the rule rather than the exception for vector-borne, and in particular tick-borne, pathogens [3–9]. Notable coinfection interactions have been documented between: bacterial genotypes (Lyme disease), bacteria and viruses (HIV), macro- and micro-parasites (helminths and bacteria), and pathogenic and endosymbiotic parasites [5,10–21]. These interactions may act as driving forces to facilitate pathogen co-existence and emergence [22]. However, disentangling to what extent parasite dynamics are driven by their individual traits, their interactions with hosts (pathogen–host), with other pathogens (pathogen–pathogen), and with tick vectors (pathogen–vector–host) remains a significant challenge. In turn, the intensity of these interactions may be sensitive to local environmental conditions, which may determine a pathogen's niche and geographical range [23].

Tick-borne pathogens offer an ideal system to study pathogen–host–vector interactions. Ticks transmit a wide array of viral, bacterial, and protozoan parasites that frequently co-circulate in a shared community of vectors and hosts [5,22,24–27]. In North America *Ixodes scapularis*, the blacklegged tick, transmits at least seven pathogens of public health concern [28–30]. Coinfections between the causative agents of Lyme disease and human babesiosis, *Borrelia burgdorferi* (*Bb*) and *Babesia microti* (*Bm*) respectively, have been studied most extensively [28–34]. The geographical distribution of Lyme disease includes the Northeast and Upper Midwest USA and continues to spread [35–37] following the expansion of *I. scapularis* over the past 30 years [38–44]. Although *Bm* is transmitted by the same vector and infects a similar host community as *Bb* [32,45], *Bm* prevalence is generally lower than *Bb* in reservoir hosts and vectors and human babesiosis is only observed in regions with the highest incidence of Lyme disease [22,36,46,47]. However, *Bm* is expanding its geographical range into populations of *Bb*-infected *I. scapularis* and, in some endemic areas, infection prevalence in ticks and hosts has been found to be equal to, or greater than, *Bb* [45,48–50].

A fuller understanding of tick-borne pathogen population dynamics requires integration of multiple components of fitness, namely: (1) pathogen–host (tick-independent) interactions influencing pathogen persistence and vertical transmission in the host; (2) pathogen–tick–host interactions influencing horizontal transmission efficiency (host to tick and tick to host); (3) pathogen–pathogen interactions, with pathogens influencing each other's persistence and transmission efficiency; and (4) pathogen–tick interactions, including vector competence as well as vertical transmission in ticks [51], not addressed here, because there is little evidence for efficient transovarial transmission in ticks with either *Bb* or *Bm* [30,52–55].

Horizontal transmission of *I. scapularis*-borne pathogens depends on the host-to-tick transmission efficiency between ticks and hosts as well as the pathogen's ability to persist in a host long enough to be transmitted from nymphal ticks of cohort 1 (that infect hosts) to larval ticks of cohort 2 (that acquire infection from hosts) [14,41,56,57]. With a focus on horizontal transmission, the lower prevalence and slower geographical spread of *Bm* has historically been attributed to the lower transmission efficiency from hosts-to-ticks as well as the more restricted host range of *Bm* [46,58–60]. We have previously identified two mechanisms enhancing *Bm* fitness. Firstly, host-to-tick transmission efficiency of *Bm* is enhanced by host coinfection with *Bb* and may increase *Bm* basic reproduction number (*R*_0_) to persistence levels under average Northeast USA ecological conditions [46]. Secondly, *Bm* vertical transmission from rodent hosts to their offspring is a vector-independent transmission pathway that may significantly enhance *Bm R*_0_ in some situations [49,61]. The co-occurrence of these within-host, between host and vector, and population-level dynamic processes with different environmental sensitivities makes testing these mechanisms empirically challenging and requires the adoption of an empirically informed modelling framework.

Here, we integrate a multi-year, multiple-location, longitudinal dataset and laboratory studies with mathematical modelling to elucidate multiple fitness components influencing coinfection dynamics between *Bb* and *Bm* in natural populations of *Peromyscus leucopus*, the most competent reservoir host for both pathogens in the eastern USA. We assess coinfection dynamics in field data using a multi-state Markov model. We apply the insights gained into parameters of a mechanistic eco-epidemiological model that incorporates interactions between *Bb* and *Bm* that affect transmission between ticks and hosts [46], and vertical transmission and overwinter persistence of *Bm* in the mouse population [61,62]. We fit this model to our empirical data using approximate Bayesian computation (ABC). We use the model to explore how the aforementioned interactions shape pathogen prevalence in mouse populations seasonally and affect the long-term viability of *Bm*. Future investigations of the environmental sensitivities of key parameters identified in this study will further guide surveillance and inform predictive models for the geographical distribution of these and other emerging tick-borne pathogens.

## 2. Methods (see electronic supplemental methods for more details)

### (a) Study animals and sample preparation

Mice and ticks were sampled on a biweekly basis from grids established at three sites in both Block Island, RI (BI) and Connecticut (CT) from May to August for three years (2014–2016). Tissue, blood, and attached ticks were collected from *P. leucopus* which were tagged and released at the site of capture. Questing *I. scapularis* nymphs were collected in each grid. Pathogen prevalence in each animal (mice and ticks) was assessed via standard qPCR [63,64]. Data from this study and from the literature were used to estimate model parameters (electronic supplementary material, tables S1 and S2).

### (b) Multi-state Markov model

We used the program Mark via the RMark interface [65–67] to fit a multi-state Markov model (MSM) [68] to the mark–recapture mouse infection state data collected from the field. For each location (BI and CT) we used records of mice sampled at two or more of the seven sample points in a given year to estimate the relative intensities of transitions between infection states: uninfected (0), *Bb* infected (1), *Bm* infected (2), or coinfected (12). Not all mice were observed at all sample points; therefore, the model also allows for mice to be alive but unobserved, or dead. The MSM model is based on the parsimonious assumption that infection states were observed without error. We did not collect data on the observation error in our study, but the qPCR assays we used are known to be highly sensitive, specific, and accurate for *Bb* and *Bm* [63,64]. See the electronic supplementary material, Text for details.

### (c) Mechanistic mathematical model

We developed a mechanistic eco-epidemiological model to examine how interactions between *Bb* and *Bm*, combined with vertical transmission of *Bm* in hosts, drive the epidemiological dynamics of both pathogens (see electronic supplementary material, text). The underlying framework is similar to existing models for *Borrelia* eco-epidemiology [e.g. 69–73]. However, in contrast to most existing models, we use a semi-discrete-time formulation to capture the complex seasonality and epidemiological dynamics of the system. In the active season of each year (spring to autumn), the system is modelled in continuous time using ordinary differential equations. In the dormant season (winter), all demographic and epidemiological processes except mortality and recovery cease and the model progresses to the beginning of the next active season in a single time-step.

The ecological component of the model describes the population dynamics of mice and ticks. Mice grow logistically throughout the active season. A specified proportion of the population present at the end of the active season survive the dormant season and form the initial population for the next cycle. The tick population is divided into larvae and nymphs. A fixed number of eggs are present at the beginning of each active season. Modelling a constant number of eggs at the beginning of each year simplifies and stabilizes the ecological dynamics and provides a single control parameter (Omega) that summarizes the suitability of the environment for the tick population. Starting on a specified day of the active season, larvae emerge from these eggs at a constant rate and quest for a host. Hosts may be mice or another unspecified host type that is not competent for *Bb* or *Bm* transmission. After encountering hosts, larvae become inactive for the remainder of the season and a proportion molt to nymphs and survive the dormant season to emerge as nymphs the following year. Larvae that do not successfully find a host by the end of the active season overwinter and a proportion survive to re-emerge the following year and continue questing. Starting on a specified day of the active season, nymphs emerge from overwinter diapause at a constant rate and quest for a host. After nymphs encounter a host, or at the end of the active season, they are removed from the model. We do not include adult ticks in the model because they are not involved in the enzootic transmission cycle of *Bb* or *Bm* (adults feed on white-tailed deer which are not competent for either pathogen).

The epidemiological component of the model describes the transmission dynamics of *Bb* and *Bm* in the mouse and tick populations (electronic supplementary material, table S3). An encounter between a tick and a mouse may result in transmission if either party is infected. The probability of transmission is modified by interactions between *Bb* and *Bm* based on empirical observations: A mouse with an existing *Bm* infection has increased susceptibility to *Bb*; a coinfected mouse has an increased probability of transmitting *Bm* to larvae but a reduced probability of transmitting *Bb* to larvae. Evidence for these interactions come from previous research [46,69,73]. *Bm* may also be transmitted vertically from an infected mouse to her offspring [49,61]. No evidence for vertical transmission of *Bb* in *P. leucopus* mice has been documented [74,75, DMT unpublished data].

In the model, mice recover from *Bb* infection at a constant rate throughout the active and dormant seasons and become susceptible again, coinfection does not affect the recovery rate, and mice do not recover from *Bm*. Although some evidence exists that recovery occurs at a low rate, life-long chronic infection is a reasonable approximation as *Bm* infection can persist on average for 9 months [61] and the life expectancy of wild *P. leucopus* is less than six months [76]. We are not aware of any evidence regarding the effect of coinfection on *Bm* recovery. Therefore, in the interests of parsimony, in the model we assume that coinfection does not affect the recovery rate. Ticks do not recover from infection with either pathogen.

### (d) Parameter estimation

We used ABC to estimate model parameters by fitting model trajectories for the mouse population, tick burden, *Bb* and *Bm* prevalence in mice and ticks to three years of field data at each location. We held the parameter values constant across all three years and let the model reach approximate steady-state before comparing trajectories. Where published or our own empirical information about parameter values was available, we incorporated this into the prior distributions (electronic supplementary material, tables S1 and S2).

### (e) Viability threshold

We examined how key parameters of the model affect the viability of *Bb* and *Bm*. We define the viability threshold of each pathogen to be the minimum tick egg density required at the beginning of each season for long-term persistence. The initial tick egg density is a strong determinant of larval and nymph population densities, and we interpret it as an indicator of the quality of the local environment. We take a pathogen to be persistent if the model initialized at demographic steady-state with very low prevalence of both pathogens converges to an asymptotic state where that pathogen is present. All code is available at https://github.com/cowparsley/borrelia-babesia-eco-epi and datafiles are available at https://doi.org/10.5061/dryad.573n5tbd3.

## 3. Results

### (a) Field data

**Figure 1.**
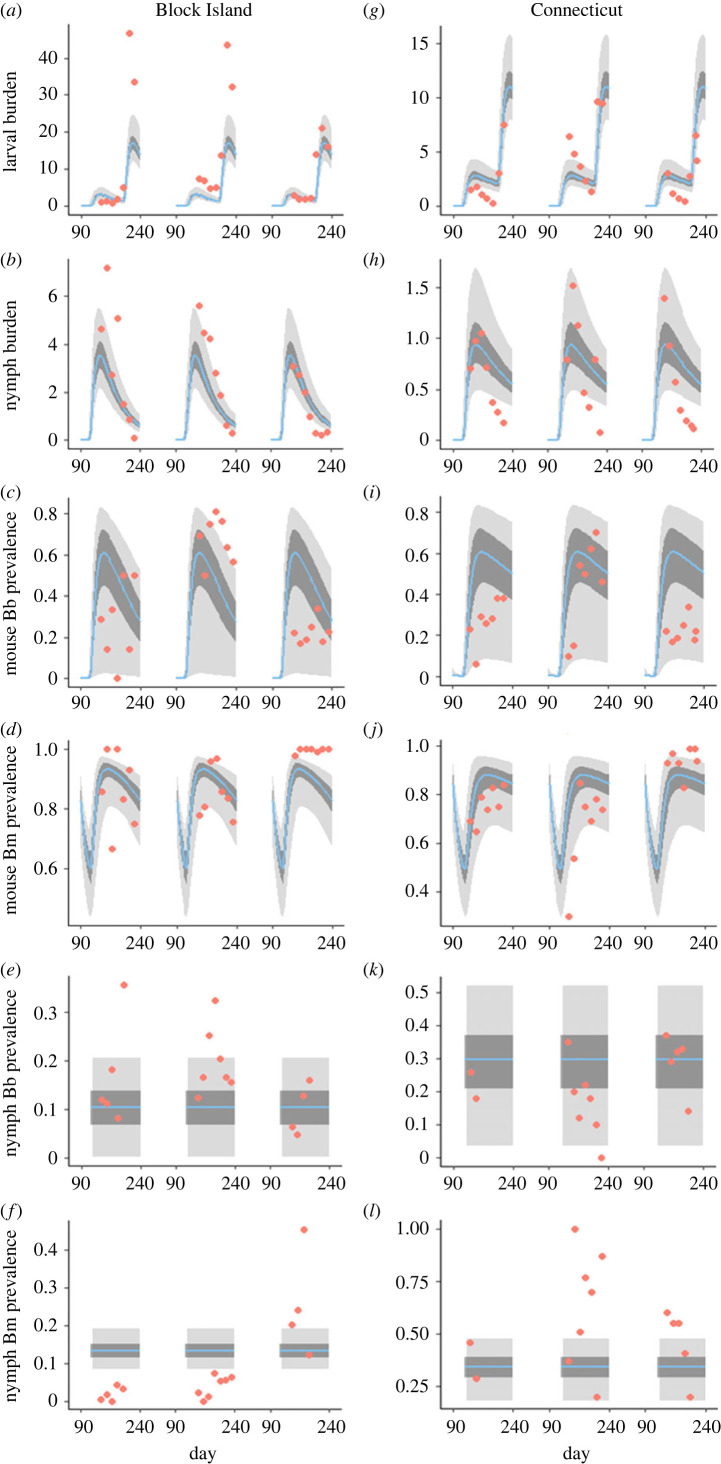
Trajectories produced by the model with parameters estimated by ABC rejection with (*a–f*) Block Island and (*g–l*) Connecticut field data. The (*a,g*) larval and (*b,h*) nymphal burden on mice, mouse infection prevalence with (*c*,*i*) *Borrelia burgdorferi* (*Bb*) or (*d*,*j*) *Babesia microti* (*Bm*), and the nymphal infection prevalence with (*e*,*k*) *Bb* or (*f*,*l*) *Bm* were calculated. Blue lines denote the model with posterior median values for each parameter. Dark grey and pale grey areas are the minimal envelope containing 1000 model trajectories with parameter values sampled from the 10% and 30% credible intervals of the posteriors, respectively. Red circles represent field data, each year is depicted with a new segment in the figure.

**Figure 2.**
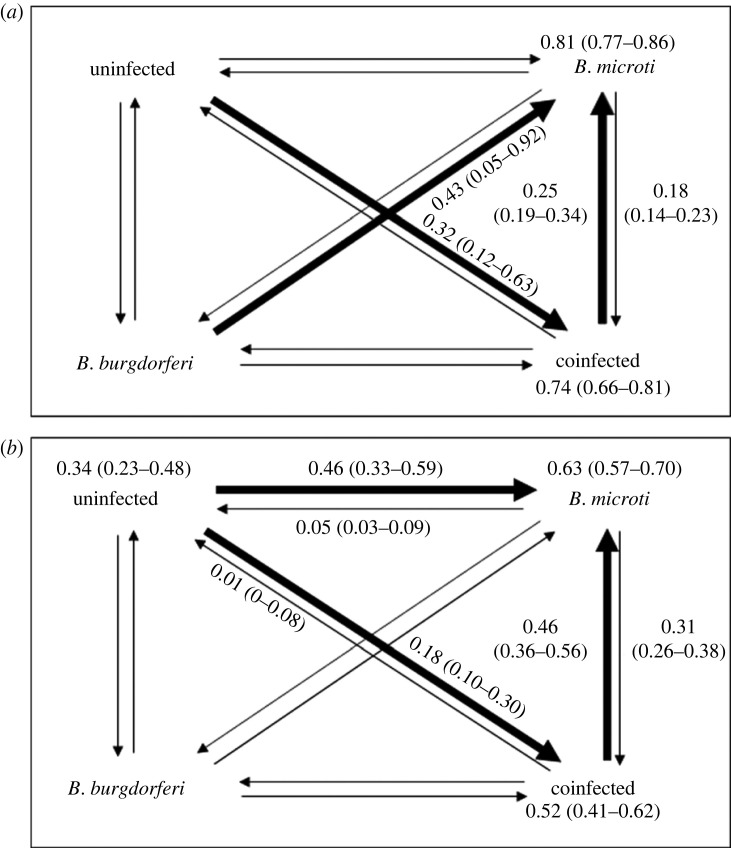
Multi-state Markov model with maximum-likelihood estimates and confidence intervals for state transition probabilities between field sessions (2 weeks) for *Peromyscus leucopus* infected mice collected from (*a*) Block Island and (*b*) Connecticut from 2014–2016. Bold arrows signify a higher probability and directional shifts. Numbers by the states are estimates that an individual will remain in those states. Low numbers of *Borrelia burgdorferi* only infected mice made it difficult to produce sensible estimates of the transition probabilities for some transitions (0.00 estimates, see electronic supplementary material, table S7 for more details).

A total of 879 *P. leucopus* mice (479 unique) from the BI sites and a total of 932 mice (535 unique) from the CT sites were included in our analyses. Observed mouse density was higher on BI compared to CT and tick burdens (larvae and nymphs) were higher from mice on BI (figure 1; electronic supplementary material, figure S1). Infection prevalence of *Bb* and *Bm* varied substantially in mice and nymphs at each site and between years (figure 1; electronic supplementary material, table S4). In mice, *Bm* was significantly more prevalent at both locations (BI and CT) compared to *Bb* in each year (all Fisher exact *p* < 0.001). A total of 1709 nymphs from BI and 1699 nymphs from CT were analysed for *Bb* and *Bm* (electronic supplementary material, table S4). In nymphs, *Bm* prevalence was significantly higher compared to *Bb* at the CT sites each year (*p* < 0.00001). However, on BI there was no significant difference between infection prevalences in 2014 (*p* = 0.869), *Bb* was significantly higher than *Bm* in 2015 (*p* < 0.00001), but *Bm* was significantly higher than *Bb* in 2016 (*p* < 0.00001) (figure 1). Coinfections between *Bb* and *Bm* were observed in mice and nymphs but were significantly more prevalent in mouse populations for all locations and years (*p* < 0.0001) except CT 2016 (*p* = 0.8834) (electronic supplementary material, table S4) (figure 2).

### (b) Multi-state Markov model

At the time of observation, 61% of mice were infected with *Bm* only, 28% were coinfected with *Bb* and *Bm*, 9% were uninfected, and 1% were infected with *Bb* only. Our primary focus was on transitions between infections states, which requires the same mouse to be observed on two or more occasions. For both the BI and CT datasets, there were 395 observations involving the same mouse at different sample points. Very few of these transitions involved mice in the uninfected or *Bb* only infected states. The MSM model analysis indicated that, between any two field sessions (approx. 2 weeks) most *Bm*-infected mice either remain in that state (probability 0.81 BI, 0.63 CT) or become coinfected (probability 0.18 BI, 0.31 CT). Most coinfected mice either remain in that state (probability 0.74 BI, 0.52 CT) or become infected with *Bm* only (probability 0.25 BI, 0.46 CT). This result indicates that recovery from *Bb* is relatively common, but recovery from *Bm* is rare, which partly motivated the representation of *Bm* infections as chronic in the mechanistic model. The small number, or absence, of observations of transitions involving the *Bb* infected state limits meaningful interpretation of the effects of facilitative or competitive interactions between *Bb* and *Bm*.

### (c) Mechanistic mathematical model

#### (i) Parameter estimation

The mechanistic model reproduces key features of the field data with reasonable parameter values. It captures the seasonal pattern of the larval and nymphal burdens on mice, including the timing and magnitude of peak burdens (figure 1). The model produces *Bb* and *Bm* infection prevalences broadly in line with previous observations, given the inter-annual variation and noise in the mouse and nymphal infection prevalence (NIP) empirical data. The parameter estimates (electronic supplementary material, table S5; electronic supplementary material, figures S2–S4) highlight several environmental differences between the two field locations. Mouse reproductive carrying capacity and tick egg density at the beginning of each season both showed higher estimated values for BI than CT for all credible intervals (electronic supplementary material, table S5). The density of non-competent hosts has a less important epidemiological role in reducing tick infection in CT than BI. Finally, estimates for the epidemiological parameters are similar for both field sites.

#### (ii) Viability threshold

**Figure 3.**
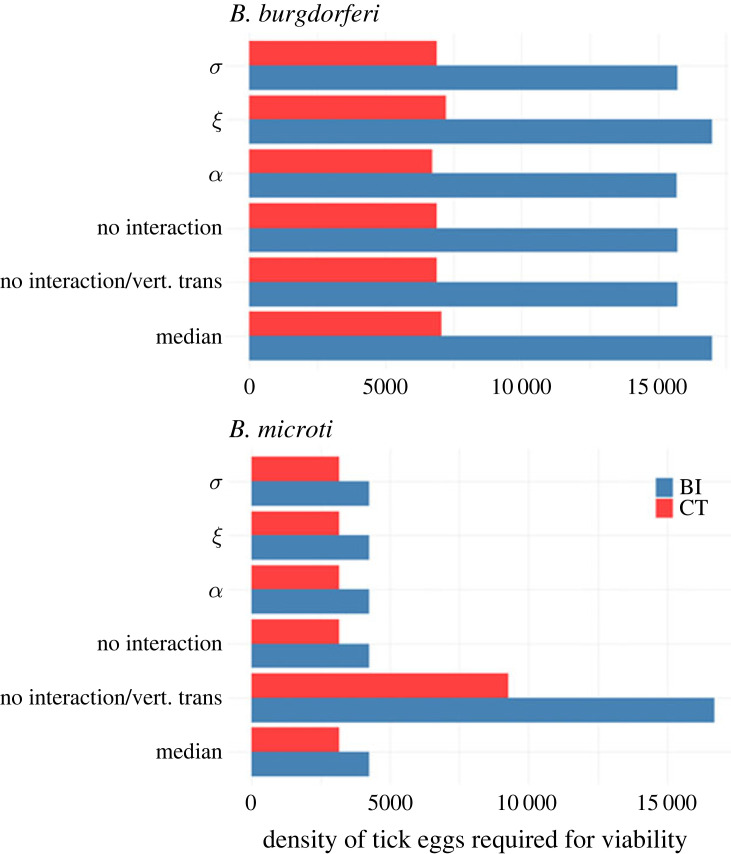
Impact of vertical transmission and pathogen interactions on the minimum density of tick eggs required at the beginning of each season for endemic persistence. Longer bars signify a larger tick population is required to support that parasite. Each pair of bars corresponds to implementation of a different set of interactions in the model: *σ*, *Bm* enhancement of *Bb* susceptibility only; *ξ*, *Bm* interference with *Bb* transmissibility only; *α*, *Bb* enhancement of *Bm* transmissibility only. No interaction: all direct interactions neutral (*α*, *ξ*, *σ* = 1). No interaction or vertical transmission: all direct interactions neutral and no vertical transmission. Median: all interactions and vertical transmission implemented with median parameter estimates for each field study location given in electronic supplementary material, table S5. These are also the parameter values used for all other scenarios, except as stated.

We calculated the minimum tick egg density, as a proxy for tick population density, required at the start of each season for each pathogen to persist. When the model is parameterized with the median values generated in the estimation process, the tick population density required for viability of *Bb* is 2.2 times larger than *Bm* at the CT sites, and 4 times larger at the BI sites (figure 3). This result suggests that the interplay of competent and non-competent host populations, and possibly factors such as overwintering survival, make the environment at the CT sites more suitable for the transmission of tick-borne infections in general, and *Bm* in particular. All the *Bb*-*Bm* interactions included in the model have a negligible impact on the viability of either pathogen at either location. However, if vertical transmission is removed from the model, persistence of *Bm* requires a 3 to 4-fold increase in the tick population density and *Bm* becomes less viable than *Bb*.

#### (iii) Seasonal prevalence trajectories derived from the mechanistic model

**Figure 4.**
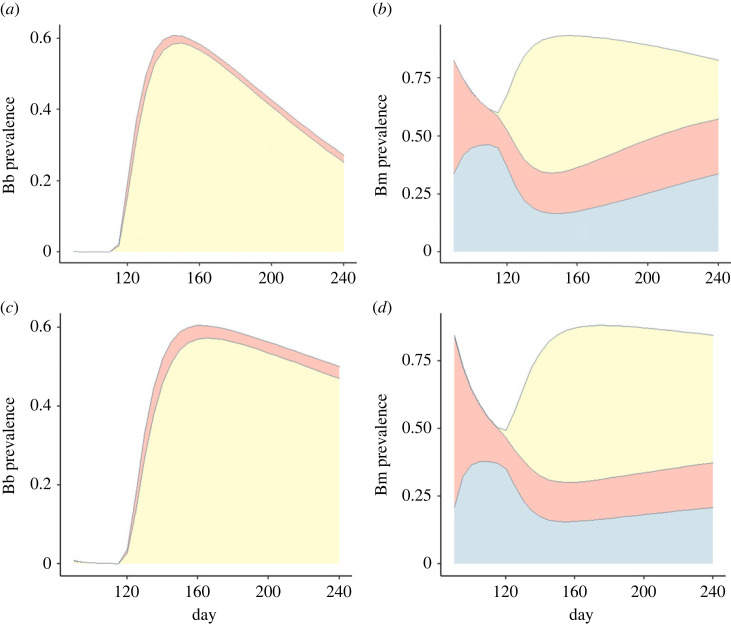
Seasonal variation of (*a*,*c*) *Borrelia burgdorferi* (*Bb*) and (*b*,*d*) *Babesia microti* (*Bm*) prevalence in mice from the mechanistic model with the median parameter values for (*a,b*) BI and (*c,d*) CT. The red-orange area denotes single infection via tick transmission (either *Bb* or *Bm*), the yellow area denotes coinfection (via any combination of transmission routes), and the blue area denotes single *Bm* infection via vertical transmission. (*b*,*d*) The red-orange and blue areas combined denote the total prevalence of single *Bm* infection.

We used the mechanistic model, parameterized with the median values generated in the estimation process, to produce approximately steady-state trajectories for *Bb* and *Bm* prevalence in mice over each year (figure 4). These trajectories offer further insight into the contrasting epidemiological drivers of the two pathogens. In the model, *Bb* prevalence is very low at the beginning of the active season but grows rapidly once nymphs emerge. Then a gradual decline in *Bb* prevalence is observed, driven primarily by recovery and mortality. Most *Bb* infections in mice occur as coinfections with *Bm*, reflecting the high prevalence of *Bm* throughout the active season. *Bm* prevalence shows a more complex annual pattern. At the beginning of the active season there is high prevalence of single *Bm* infections due to persistent infection in the overwintered population and ongoing vertical transmission. The prevalence of single infections arising from tick-borne transmission in the previous season decreases quickly due to mortality, but the prevalence of single infections from vertical transmission increases due to a burst of rapid mouse population growth, slowing as the population approaches carrying capacity. At the same time, the emergence of nymphs and intense *Bb* transmission rapidly converts single *Bm* infections to coinfections. Mortality and recovery drive a gradual decline in the prevalence of coinfections and increase in the prevalence of single *Bm* infections while ongoing mouse reproduction drives a gradual increase in the prevalence of single *Bm* infections due to vertical transmission (figure 4; electronic supplementary material, figure S5).

#### (iv) Sensitivity analysis

The sensitivities of infection prevalence variables (electronic supplementary material, figures S6 and S7) and of demographic variables (electronic supplementary material, figure S8) were calculated and key parameters related to mouse and nymph infection were determined.

## 4. Discussion

Despite extensive recognition of the importance of coinfection in driving parasite dynamics [5,22,77–79], quantifying the roles of individual parasite traits versus ecological interactions in shaping parasite population dynamics in natural systems remains a challenge [80,81]. Here we integrated data from field and laboratory studies into mathematical models to disentangle the multiple fitness components influencing the population dynamics of emerging tick-borne pathogens. We found that the importance of different transmission pathways varied seasonally and the models indicated that epidemiological dynamics are dominated by the individual trait of vertical transmission of *Bm* and are less sensitive to pathogen-pathogen interactions than previously suspected on the basis of *R*_0_ models that do not account for *Bm* vertical transmission [46].

Analysis of our field data using a multi-state Markov model indicated that recovery from *Bb* is relatively common, but recovery from *Bm* is rare. The prevalence of *Bm* infection was higher compared to all other states of infection and uninfected mice, while *Bb*-only infected mice were rare. Most mice observed early in the trapping season (April and May) were already infected with *Bm*, which limited our ability to investigate relative transmission efficiencies or pathogen facilitation. This early season infection may have resulted from vertical transmission, or from mice coming into contact with infected nymphs before sampling began. Our models were unable to account for temporal variation in the number of nymphs each host was exposed to. However, given the abundance of nymphs in the environment and their ability to attach to hosts frequently, the removal of ticks at one time point seems to have a limited effect on transmission probabilities at subsequent time points. Additionally, while co-feeding transmission may be important for some systems, peak activity of immature life stages at our sampling locations are highly asynchronous, limiting the importance of co-feeding on transitions between infection states at our study locations [56,57,82].

The epidemiological model with the median posterior parameter values produced steady-state trajectories that captured the main qualitative aspects of the field data and were in reasonable quantitative agreement, except for *Bm* prevalence in nymphs. Nevertheless, our field observations contain associations that are difficult to rationalize, in particular the intra-annual variation in NIP and the persistent saturation of *Bm* in mice in 2016. These divergencies may be due to substantial noise in the data, along with other factors. Our field observations found that *Bm* prevalence is high in mice, but low in nymphs. These patterns are consistent with weak transmission efficiency from mice to larvae [46,59] along with vertical transmission in mice [49,61]. Nevertheless, in 2016 (figure 1*d,j*), *Bm* prevalence in mice was close to 100% in both regions for most of the active season. The model trajectories suggest that, in the latter part of the active season, *Bm* prevalence in mice should gradually decline in mice because vertical transmission is imperfect and the questing nymph population is small. Therefore, it is not clear how such high prevalence is maintained in natural populations.

Our field observations also showed substantial intra-annual NIP variability, even though we expect within-season NIP to remain constant because almost all infections occur as the result of larval transmission in the previous year. It is possible that this variability is a consequence of the sample size; although we collected approximately 500 nymphs from each location each year, this represents a small fraction of the total population. Additionally, overwintered spring larvae would host-seek, feed, and molt at the end of the nymphal season. Because these late host-seeing nymphs fed on a different cohort of hosts than those the previous year, infection prevalence may vary and partially explain the observed intra-annual differences in NIP [83]. Studies of physiological age [84] could help determine the extent to which a nymphal cohort is composed of larvae fed the same year (spring) or the previous (summer).

Other longitudinal field studies have shown significant inter-annual variation in pathogen prevalence possibly resulting from environmental factors, such as temperature affecting questing tick activity, density and emergence, vector and host density, tick burdens, and habitat or vegetation type [85,86]. In mice, intra-annual variability in *Bb* prevalence is expected because mice may clear infection and become subsequently reinfected over the season. Variability of *Bm* can be derived from different transmission pathways, vertical transmission early in the season and vector-mediated infection later in the season [61].

The role of vertical transmission on *Bm* persistence is illustrated by the endemic threshold analysis. At very low levels of *Bm* vertical transmission, the viability analysis indicates that *R*_0_ is higher for *Bb* than for *Bm*. This is consistent with previous findings that the distribution of *Bm* is restricted to highly endemic areas for *Bb* [22,36,46,47]. However, even moderate levels of vertical transmission reverse the order, resulting in higher *R*_0_ values for *Bm* compared to *Bb*. This reversal is consistent with the high prevalence of *Bm* reported in some *Bb* endemic areas, including the field data presented in this study [45,48–50]. However, it is in clear contrast to reports from other regions where *Bb* is present and *Bm* is absent [87]. Further research on the environmental sensitivity of vertical transmission, particularly in the overwintering mouse population, is needed to understand and predict its effect on the geographical distribution of *Bm*.

Our study contributes to a more robust understanding of the mechanisms driving the infection and coinfection dynamics of *Bb* and *Bm*. Further studies should focus on elucidating environmental sensitivities and ecological factors driving the distribution and abundance of *Bb* and *Bm* to understand geographical spread and identify thresholds for control of tick-borne pathogens [88].

## Data Availability

Code is available in the supplemental materials document and on GitHub (see https://github.com/cowparsley/borrelia-babesia-eco-epi). Data files are available from Dryad [89]. Additional information is provided in electronic supplementary material [90].
